# Lifestyle Modifications and Nutritional Interventions in Aging-Associated Cognitive Decline and Alzheimer’s Disease

**DOI:** 10.3389/fnagi.2019.00369

**Published:** 2020-01-10

**Authors:** Gurjit Kaur Bhatti, Arubala P. Reddy, P. Hemachandra Reddy, Jasvinder Singh Bhatti

**Affiliations:** ^1^Department of Medical Lab Technology, University Institute of Applied Health Sciences, Chandigarh University, Mohali, India; ^2^Department of Pharmacology and Neuroscience, Texas Tech University Health Sciences Center, Lubbock, TX, United States; ^3^Internal Medicine, Texas Tech University Health Sciences Center, Lubbock, TX, United States; ^4^Neuroscience and Pharmacology, Texas Tech University Health Sciences Center, Lubbock, TX, United States; ^5^Neurology, Departments of School of Medicine, Texas Tech University Health Sciences Center, Lubbock, TX, United States; ^6^Public Health Department of Graduate School of Biomedical Sciences, Texas Tech University Health Sciences Center, Lubbock, TX, United States; ^7^Speech, Language and Hearing Sciences Department, School Health Professions, Texas Tech University Health Sciences Center, Lubbock, TX, United States; ^8^Department of Biotechnology and Microbial Biotechnology, Sri Guru Gobind Singh College, Chandigarh, India

**Keywords:** Alzheimer’s disease, oxidative stress, inflammation, diet, exercise, lifestyle, nutraceuticals, antioxidants

## Abstract

Alzheimer’s disease (AD) is a type of incurable neurodegenerative disease that is characterized by the accumulation of amyloid-β (Aβ; plaques) and tau hyperphosphorylation as neurofibrillary tangles (NFTs) in the brain followed by neuronal death, cognitive decline, and memory loss. The high prevalence of AD in the developed world has become a major public health challenge associated with social and economic burdens on individuals and society. Due to there being limited options for early diagnosis and determining the exact pathophysiology of AD, finding effective therapeutic strategies has become a great challenge. Several possible risk factors associated with AD pathology have been identified; however, their roles are still inconclusive. Recent clinical trials of the drugs targeting Aβ and tau have failed to find a cure for the AD pathology. Therefore, effective preventive strategies should be followed to reduce the exponential increase in the prevalence of cognitive decline and dementia, especially AD. Although the search for new therapeutic targets is a great challenge for the scientific community, the roles of lifestyle interventions and nutraceuticals in the prevention of many metabolic and neurodegenerative diseases are highly appreciated in the literature. In this article, we summarize the molecular mechanisms involved in AD pathology and the possible ameliorative action of lifestyle and nutritional interventions including diet, exercise, Calorie restriction (CR), and various bioactive compounds on cognitive decline and dementia. This article will provide insights into the role of non-pharmacologic interventions in the modulation of AD pathology, which may offer the benefit of improving quality of life by reducing cognitive decline and incident AD.

## Introduction

Due to improved healthcare infrastructures and early diagnosis of diseases, human life expectancy is increasing globally (Oeppen and Vaupel, 2002). However, the burden of age-associated diseases will also increase exponentially in the coming decades (Beard et al., 2016). Aging is a process that is regulated by various genetic and molecular mechanisms. Dementia is one of the major causes of disability in the aging population. Alzheimer’s disease (AD), the most dominant form of dementia, accounting for 60–80% of dementia cases, is a multifactorial neurodegenerative disease characterized by the accumulation of amyloid-β (Aβ; plaques) outside neurons and the hyperphosphorylation of tau protein and neurofibrillary tangles (NFTs) inside the neurons in the brain, which lead to cognitive deficit, memory loss, and then neuron death. With the aging of the population, the burden of age-related neurodegenerative disorders is increasing at an exponential rate all over the world (Prince et al., 2015). Multiple risk factors including age, education, socioeconomic status, family history, gene mutations, oxidative damage, neuroinflammation, et cetera are involved in the pathophysiology of AD. Currently, pharmacologic drugs targeting Aβ and tau proteins are unsuccessful in clinical trials, and there is no treatment available for the cure of AD to date. Due to the failure of pharmacologic treatments for AD, non-pharmacological interventions have gained more attention for the prevention of this disease. Many common molecular mechanisms are shared among the metabolic and neurodegenerative disorders, which include mitochondrial dysfunction, oxidative stress, inflammatory pathways, and calcium homeostasis. There are many preventive strategies that target these pathways and are found to be very effective in limiting the burden of metabolic diseases such as diabetes, obesity, cardiovascular diseases (CVDs), and cancer. In this review article, we focus on the molecular mechanisms involved in AD and the lifestyle and nutritional interventions, such as physical exercise, a Mediterranean diet (MD), nutraceuticals, and bioactive compounds, as preventive strategies for the development of AD in the elderly population. These interventions for the prevention or delay of the onset of dementia could have significant effects on individuals, society, and healthcare providers.

## Prevalence and Economic Burden of Alzheimer’s Disease

**Figure 1:**
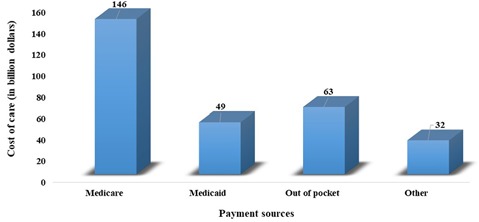
Annual expenditure, including health care, long-term care, and hospice care for people with dementia.

Current demographic trends indicate a significant increase in older people (above 65 years) in the world’s population, posing a major risk for the development of dementia. A previous study indicates that about 6% of the world population is suffering from dementia (Prince et al., 2013). Recent estimates by the Alzheimer’s Association reveal that globally, 55 million people have dementia and every year, more than 10 million people develop this deadly disease. This number is predicted to increase by 88 million in the year 2050 (Alzheimer’s Association, 2019). This disease is more prevalent in Americans. Currently, 5.8 million Americans of all ages were living with Alzheimer’s dementia in 2019, and this number is projected to increase by three folds by the year 2050. About 5.6 million people are above 65 years old. Every 10th American aged above 65 years is suffering from AD. This increase in the incidence and prevalence of AD in the elderly population has become a major public health challenge associated with an economic burden on individuals, society, caregivers, and federal governments (Hurd et al., 2013). Figure 1 shows the annual expenditure, including for health care, long-term care, and hospice care for people with dementia. Recent estimates predict that there will be a heavy increase in the overall maintenance cost of AD, which is expected to increase from $290 billion in 2019 to more than $1.1 trillion in 2050 (Alzheimer’s Association, 2019). However, reducing the potentially modifiable risk determinants, including educational status, cigarette smoking, sedentary lifestyle, depression, hypertension, diabetes, dyslipidemia, and obesity, may help in the prevention of about 500,000 cases of dementia in the United States (GBD 2015 Neurological Disorders Collaborator Group, 2017).

## Molecular Mechanisms Involved in Alzheimer’s Disease Pathology

### Amyloid β Plaques and Tau Hyperphosphorylation

Two major proteins in the brain are involved in the pathophysiology of AD, i.e., the Aβ and tau proteins. The Aβ protein, consisting of 39–43 amino acid residues, is produced intracellularly in the brain. A disparity between the accumulation and clearance of Aβ leads to plaque formation in the brain. It plays a vital role in the progress of AD pathology and cognitive impairment (Hardy, 1997; George-Hyslop and Rossor, 2001). Genetic mutations in the genes encoding Aβ, Aβ precursor protein (AβPP), and Presenilins (PS1 and PS2) lead to abnormal Aβ aggregation in the brain. In AD, the build-up of amyloid fibrils as amyloid plaques or senile plaques in the extracellular region of brain cells is responsible for synaptic damage, neuronal dysfunction, and inflammatory responses (Lesné et al., 2013). Tau protein, a family of natively unfolded microtubule-associated proteins, is located on chromosome 17q21 and plays an important role in microtubule assembly and stabilization. In AD pathology, the intense hyperphosphorylation of tau protein causes the formation of NFTs, leading to microtubule disassembly and neuronal loss in the area of the brain associated with memory and learning centers (Kolarova et al., 2012).

### Mitochondrial Dysfunctioning

**Figure 2:**
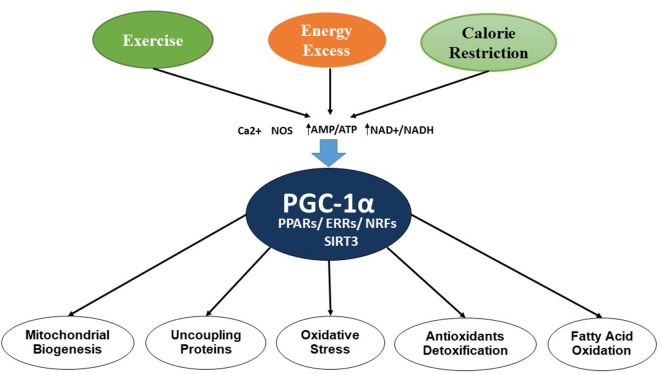
Control of mitochondrial biogenesis by transcription factors and PGC-1α.

Mitochondria are double-membrane, intracellular organelles present in the cells and are known to play a vital role by metabolizing nutrients. They are also known as the powerhouse of the cell or “energy currency,” as they generate adenosine triphosphate (ATP). Several studies have implicated mitochondrial dysfunction as a major pathologic condition involved in neurodegenerative diseases. Mitochondrial dysfunction involves alterations in the processes of mitochondrial biogenesis and dynamics, which leads to many pathologic conditions. In mitochondrial biogenesis, the number and size of mitochondria increases, which is controlled by peroxisome proliferator-activated receptor (PPAR)-γ coactivator-1α (PGC-1α), involving several transcription factors and other proteins including nuclear respiratory factors (NRF-1 and NRF-2), uncoupling proteins (UCP2), transcription factor A (Tfam), PPARs, thyroid hormone, glucocorticoid, estrogen, and estrogen-related receptors (ERR) α and γ (Hock and Kralli, 2009). AMP-activated protein kinase (AMPK) also contributes to the regulation of intracellular energy metabolism (Reznick and Shulman, 2006; Bhatti et al., 2017a). Figure 2 shows the regulation process of mitochondrial biogenesis by various transcription factors and other proteins.

Mitochondrial dynamics is a process by which mitochondria maintain their shape, structure, and functions by continuously going through the fission and fusion process (Chan, 2006; Westermann, 2010; Archer, 2013; Roy et al., 2015). In mitochondrial fusion, three GTPase genes, including Mitofusin 1 and 2 and optic atrophy1 (Opa1), regulate this process, whereas the mitochondrial fission is controlled by two GTPase genes, Fis1 and Drp1. Impaired mitochondrial biogenesis and dynamics lead to disturbed normal functioning of mitochondria, resulting in diminished energy generation in the cells. It is evident from previous studies that impaired mitochondrial dynamics plays a vital role in aging and aging-associated metabolic and neurodegenerative diseases (Reddy, 2011). During mitochondrial biogenesis, some defective mitochondria that are formed are then removed by a process called mitophagy. The defective mitochondria fuse with the lysosomes and are removed by the autophagy-lysosome system (Ding and Yin, 2012). Aging contributes to the accumulation of defective mitochondria, oxidative imbalance, and apoptosis by impairing mitophagy (Chistiakov et al., 2014). Modifications in mitochondrial biogenesis and dynamics also lead to the overproduction of reactive oxygen species (ROS) in the cells, ultimately causing oxidative damage. Autophagy is a lysosome-mediated degradative pathway that facilitates the elimination of defective organelles and recycles various cellular components, including lipids and proteins (Shintani and Klionsky, 2004). Impairment in autophagy may lead to the accumulation of Aβ protein in disease conditions. Autophagy deficit may contribute to age-associated neurodegenerative diseases, including AD (Martinez-Vicente, 2015; Zare-Shahabadi et al., 2015). Emerging strands of evidence indicate that the disruption in the mammalian target of rapamycin (mTOR) signaling pathway impacts multiple cellular functions, including autophagy, glucose metabolism, cell growth, and mitochondrial functions, that are central in aging and neurodegenerative diseases (Perluigi et al., 2015). This compelling evidence indicates that targeting mTOR in the brain might be another promising strategy that could enable drug discovery for AD. The dysregulation of the PI3K/AKT/mTOR pathway and autophagy defects in the brains of AD patients might be targeted for the development of new drugs. Reddy and Oliver (2019) recently demonstrated that the accumulation of Aβ and phosphorylated tau induces defective autophagy and mitophagy in AD.

**Figure 3:**
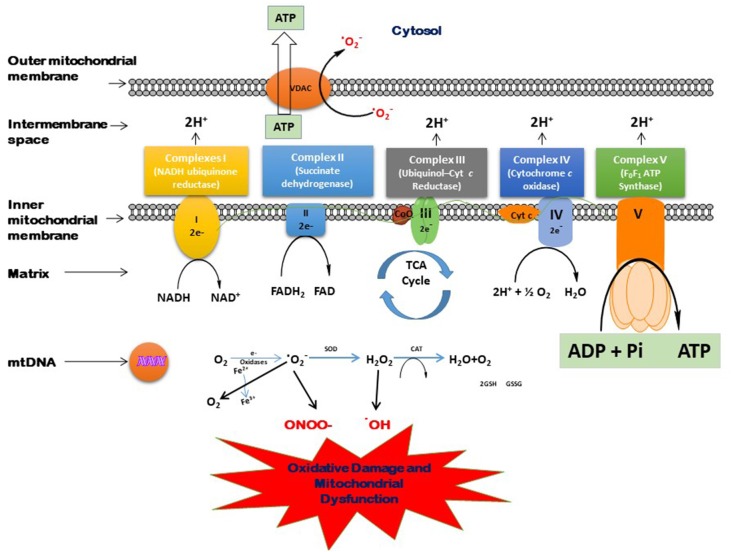
Process of free radical generation and adenosine triphosphate (ATP) biosynthesis in the cell.

With advanced age, the oxidative damage induced by excessive generation of free radicals reduces antioxidant capacity, and proinflammatory reactions lead to the aging-related pathologic conditions. The brain is very much affected by these oxidative biomarkers. Moreover, the brain normally has less oxidant capacity than other organs. In dementia, the accumulation of neurotoxic peptides such as Aβ and tau might damage the brain tissues (Kapogiannis and Mattson, 2011; Mao and Reddy, 2011). Mitochondria play a vital role in several metabolic processes. The modifications in the mitochondrial structure and function may lead to several age-associated neurodegenerative diseases (Reddy, 2006, 2011; Roy et al., 2015). The generation of various ROS and their scavenging is a routine function that takes place in the mitochondria. An imbalance between the generation of free radicals in the cells and the ability to detoxify is called oxidative stress. In the Kreb’s cycle, the electrons are contributed by NADH and FADH_2_. These electrons are then transferred through the electron transport chain (ETC), which generates electrochemical gradient across the inner mitochondrial membrane and then produces energy in the form of ATP (Andreyev et al., 2005). Figure 3 shows the process of the generation of free radicals and ATP biosynthesis in the cell. However, this process also leads to the excessive generation of several reactive species such as superoxide anion (^•^O_2_), hydroxyl radical (^•^OH), nitric oxide (NO), and reactive nitrogen species (Dröge, 2002; Valko et al., 2006). The overproduction of these ROS may damage proteins, lipids, and DNA (Beckman and Ames, 1999), which disrupts ATP biosynthesis and other functions in mitochondria (Dröge, 2002; Murphy, 2009). Cells tend to neutralize the oxidative damage induced by the overproduction of ROS either by enzymatic or non-enzymatic mechanisms. The main enzymes known to detoxify ROS are superoxide dismutase (SOD), catalase (CAT), glutathione reductase (GR), and glutathione peroxidase (GPx). On the other hand, there are many non-enzymatic mechanisms that protect the cells against oxidative damage, including glutathione (GSH), vitamins E and C, carotenoids, polyphenols, and flavonoids.

### Neuroinflammation

AD pathology is not restricted to the aggregation of misfolded amyloid or tau proteins; some immunological mechanisms are also involved in the brain. Neuroinflammation induced by these misfolded proteins is another key hallmark of AD and might be targeted as a therapeutic strategy along with peptides (Heneka et al., 2015). These misfolded proteins, including Aβ plaques and NFTs in the brain, initiate innate immune responses by interacting with toll-like receptors (TLRs) and CD4 cells. There is substantial evidence for neuroinflammation in the early stages of AD development (Yasuno et al., 2008). Recent studies also established the role of variants of many immune receptor genes, including TREM2 and CD33, in the pathogenesis of AD (Griciuc et al., 2013; Guerreiro et al., 2013; Jonsson et al., 2013). Excesses of free radicals, NO, cytokines, and some proteolytic enzymes may be responsible factors that are associated with neuroinflammation and may promulgate neuronal death (Cherry et al., 2014; Yuste et al., 2015). All of these measures are crucial in age-associated cognitive decline and AD pathology.

### Epigenetic Control of Neurodegenerative Diseases

The term epigenetics refers to mitotically and meiotically heritable changes in gene expression in response to environmental stimuli, including stress, diet, or exposure to adverse environmental factors, without altering DNA sequences (Babenko et al., 2012; Griñan-Ferré et al., 2016). The main epigenetic mechanisms are DNA methylation, histone post-translational modifications, and the regulation of gene expression mediated by noncoding RNA molecules (Moore et al., 2013; Holoch and Moazed, 2015; Hwang et al., 2017). DNA methylation is a well-known epigenetic mechanism that involves the addition of a methyl group onto the C5 position of the cytosine to form 5-methylcytosine with the help of an enzyme, a DNA methyltransferase. DNA methylation regulates gene expression by recruiting proteins involved in gene repression or by inhibiting the binding of transcription factor(s) to DNA (Moore et al., 2013). Histones are the most abundant proteins associated with DNA and aggregate with each other, forming the histone octamer around which DNA is wrapped to create the nucleosome (Bannister and Kouzarides, 2011). The N-terminal tails of histones may undergo several post-translational modifications, including acetylation, methylation, phosphorylation, ubiquitination, and ADP ribosylation. These changes influence the chromatin structure, facilitating or inhibiting gene transcription (Bannister and Kouzarides, 2011). In addition to DNA methylation and histone modifications, the regulation of gene expression mediated by noncoding RNA molecules occurs in many tissues (Peschansky and Wahlestedt, 2014; Holoch and Moazed, 2015).

Global DNA modification studies have highlighted a potential role for epigenetic mechanisms in the complex etiology of various neurodegenerative diseases, particularly AD (Bradley-Whitman and Lovell, 2013; Coppieters et al., 2014; Roubroeks et al., 2017). Besides the nuclear DNA, there is growing evidence that the mitochondrial DNA (mtDNA) could be controlled by epigenetic mechanisms (Hroudová et al., 2014; Blanch et al., 2016; Stoccoro et al., 2017). Several studies have demonstrated the impact of epigenetic modifications on the pathogenesis of neurodegenerative diseases (Urdinguio et al., 2009; Gruber, 2011; Gangisetty and Murugan, 2016; Bassi et al., 2017; Smith and Lunnon, 2017; Berson et al., 2018; Gangisetty et al., 2018; Lardenoije et al., 2018; Lascano et al., 2018; Qazi et al., 2018; Stoccoro and Coppedè, 2018). There is a growing body of evidence suggesting that epigenetic mechanisms mediate the risk for AD. Intense research in experimental models suggests that molecular interventions for modulating epigenetic mechanisms might have therapeutic applications to promote cognitive maintenance to an advanced age (Griñan-Ferré et al., 2016).

### Modifiable Risk Factors

**Figure 4:**
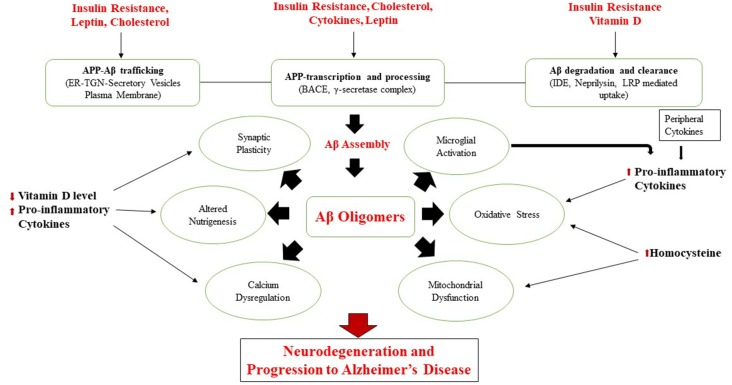
Various modifiable risk determinants in Alzheimer’s disease (AD) pathology.

Emerging evidence suggests that traditional cardiometabolic risk factors such as a sedentary lifestyle, central obesity, dyslipidemia, insulin resistance, hypertension, diabetes, and CVDs are associated with the progress of cognitive decline and AD (Cholerton et al., 2013; de la Torre, 2013; Chen et al., 2014; Geijselaers et al., 2015; Xu et al., 2015; Tamarai et al., 2019). Conversely, Calorie restriction (CR), antioxidant-rich dietary components, and certain dietary patterns may limit the progress of metabolic and neurodegenerative diseases (Everitt et al., 2006; Calder et al., 2011). The molecular mechanisms linking these modifiable factors are already discussed in our previous study (Bhatti et al., 2017b). Figure 4 shows various modifiable risk determinants affecting various molecular mechanisms in AD pathology, as demonstrated in a previous study (Chakrabarti et al., 2015).

## Pharmacologic Treatments for Alzheimer’s Disease

Many experimental treatments currently undergoing clinical trials are targeting the molecular mechanisms of AD, including Aβ plaques, tau hyperphosphorylation, oxidative damage, mitochondrial dysfunction, neurotransmission, calcium homeostasis, cell signaling, and anti-inflammatory pathways (Arvanitakis et al., 2008; Leoutsakos et al., 2012; Latta et al., 2015; Bhatti et al., 2017a; Hsu and Marshall, 2017). However, effective pharmacologic treatment strategies for cognitive decline, mild cognitive impairment (MCI), or dementia are not available to date. The accumulation of Aβ peptide and tau hyperphosphorylation are the major hallmarks of AD (Reddy and Oliver, 2019). Several clinical trials of the drugs targeting Aβ peptide and tau hyperphosphorylation failed to demonstrate any positive result. A recent systematic review of 51 unique trials from January 2009 to July 2017 rated as having low to moderate risk of bias established that the currently available drugs for dementia, hypertension, and diabetes, that is, anti-inflammatory medications such as nonsteroidal anti-inflammatory drugs, statins, and estrogen/progestin agents, neither improved nor slowed cognitive decline in persons with normal cognition or with MCI (Fink et al., 2018). This evidence shows the limited scope of these pharmacologic management approaches for cognitive protection in individuals with normal cognition or MCI. Recent studies of clinical trials focusing on cognitive training (11 trials) and physical exercise (16 trials) did not show satisfactory results for the prevention of cognitive decline or dementia (Brasure et al., 2018; Butler et al., 2018). Interestingly, a randomized controlled trial involving the multidomain intervention of exercise, diet, and cognitive training prevented cognitive decline in at-risk older people (Ngandu et al., 2015).

## Non-pharmacologic Interventions in AD Pathology

Since no effective pharmacological treatment is available to cure dementia, a greater emphasis has been placed on the implementation of non-pharmacological interventions that may prevent AD or reduce the escalation of AD burden. It is evident from various animal and human observational studies that non-pharmacologic interventions including physical exercise, CR, antioxidant supplements, diet, nutraceuticals, and several plant-based bioactive compounds are effective in reducing the modifiable risk factors such as obesity, diabetes, CVDs, cancer, etc. Many dietary interventions are known to improve insulin sensitivity, which further reduces inflammation and improves cognitive functions (Bayer-Carter et al., 2011; Kelly et al., 2011). Interventions that target modifiable risk factors for AD hold promise for reducing the incidence of AD (Xu et al., 2015). The lifestyle interventions appear to reduce the morbidity and mortality in aging populations by modulating various molecular mechanisms and might be promising non-therapeutic measures for various metabolic and aging-associated diseases (Norton et al., 2014). Recent studies suggest that elevated incidence and prevalence of cognitive decline and AD might be reduced through effective strategies targeting various cardiometabolic risk factors, including sedentary lifestyle, smoking, midlife hypertension, midlife obesity, and diabetes (Norton et al., 2014). Thus, lifestyle and nutritional intervention may be effective primary prevention strategies for AD. The possible mechanisms mediating the impact of lifestyle and nutritional interventions on cognitive decline and AD are discussed here.

## Dietary Interventions

Healthy nutritive food components, rich in their antioxidant and anti-inflammatory properties, are known to regulate the immune system and may modify the neuroinflammatory events involved in the progression of cognitive impairment and AD (McGrattan et al., 2019). These nutraceuticals and dietary patterns may constitute promising approaches in the prevention of cognitive decline or delaying the progression to AD (Canevelli et al., 2016). Several dietary components, such as omega-3 fatty acids, nutraceuticals, minerals, micronutrients, and vitamins have been examined for their roles in health and disease (Wilson et al., 2017). These dietary interventions are known to play an ameliorative role in the pathophysiology of diabetes, obesity, CVDs, and cancer, etc. The dietary interventions modulate the molecular mechanisms, including Aβ formation, tau hyperphosphorylation, oxidative stress, and epigenetic controls, in age-associated neurodegenerative diseases. Diet can modify the epigenetic mechanisms by regulating DNA methylation, acetylation, histone modifications, and changes in miRNA expression, thereby influencing the expression of particular genes responsible for epigenetic alterations (Park et al., 2012; Abdul et al., 2017).

### Polyunsaturated Fatty Acids (PUFAs)

Lipids are an essential component of the brain, wherein about one-third are essential Polyunsaturated fatty acids (PUFAs; Benatti et al., 2004). They constitute vital components of neuronal cell membranes and are involved in membrane fluidity, allowing for optimal communication between cells, cell signaling, and neuroprotection (Bazan, 2005). Essential PUFAs play a critical role in brain development and functions, with antioxidant, anti-excitotoxic, and anti-inflammatory activities. Abnormalities in PUFA status have been implicated in neuropsychiatric health and diseases, including AD (Liu et al., 2015). Many studies involving omega-3 fatty acids concerning cognitive decline have been carried out, and they show conflicting results. The long-chain omega-3 PUFAs have been shown to be involved in lowering the risk of cognitive impairment in individuals without dementia (Fotuhi et al., 2009). However, the results of other clinical trials were less conclusive. Thomas et al. (2015) recently summarized the findings of controlled studies carried out over the past 10 years and suggested that omega-3 fatty acid supplementation is advantageous only in the initial stages of cognitive decline. Another study demonstrated that fish intake (≥100 g/week) might slow the progress of cognitive decline in the Chinese population (>65 years; Qin et al., 2014). Another randomized clinical trial involving supplementation of omega-3 PUFA to participants aged 60 years without dementia or cognitive impairment showed no significant improvement in cognitive function (Sydenham et al., 2012). Recently, a randomized, placebo-controlled trial reported that long-term use of omega 3 PUFA supplementation with or without multidomain intervention had no significant impact on the cognitive decline over 3 years (Andrieu et al., 2017).

### Curcumin

Curcumin is isolated from the rhizome of *Curcuma longa*, produced mainly in India and China (Ammon et al., 1992). It is the principal active compound of turmeric, an Asian spice, and is known to play a key role in disease prevention through the modulation of various biochemical pathways (Prasad et al., 2014; Kunnumakkara et al., 2017). Turmeric powder is used as a traditional medicine against many conditions because of its antioxidant, anti-inflammatory, antibacterial, antiviral, antifungal, and anticancer activities (Sikora et al., 2010; Rahmani et al., 2018). Frequent use of curcumin in curry may be associated with better cognitive performance and low prevalence of AD in elderly Indian populations compared with the US population (Ganguli et al., 2000; Ng et al., 2006). Recent studies have established that curcumin plays a protective role against Aβ in AD due to its potent antioxidant, anti-inflammatory, and neuroprotective actions (Sundaram et al., 2017; Reddy et al., 2018). A randomized, placebo-controlled, double-blind, clinical trial of curcumin (1–4 g/day) in 34 AD patients shows no significant effect (Baum et al., 2008). Similarly, another randomized clinical trial of Curcuminoids (2 or 4 g/day) in 36 patients with dementia did not show any significant effect (Ringman et al., 2012). Curcumin significantly downregulated the expression of class I HDACs (HDAC1, HDAC3, and HDAC8) and upregulated the acetylated histone H4 levels in Raji cells, thereby modulating the epigenetic control (Liu et al., 2005). Curcumin has been shown to inhibit certain epigenetic enzymes (Reuter et al., 2011; Vahid et al., 2015). The results of several studies indicated that although curcumin has very strong neuroprotective properties, its bioavailability needs to be improved for future therapeutic strategies against neurodegenerative diseases.

### Flavonoids

Flavonoids are natural compounds with a polyphenolic structure and are commonly found in fruits, vegetables, grains, bark, roots, stems, flowers, tea, and wine (Panche et al., 2016). According to their chemical composition, flavonoids are categorized into various subclasses such as flavonols, flavones, flavanones, flavanols, anthocyanins, isoflavones, chalcones, and dihydrochalcones. Several studies have suggested that flavonoids display strong antioxidative, anti-inflammatory, anti-mutagenic, and anti-carcinogenic properties (Pietta, 2000; Panche et al., 2016). Due to these properties, the flavonoids play a preventive role in the pathology of cancer, Alzheimer’s, and CVDs (Benavente-García and Castillo, 2008). The flavonoids possess an ability to reduce the expression of pro-inflammatory cytokines, modulate epigenetic control, down-regulate inflammatory biomarkers, and prevent neural damage and many other diseases, mainly due to their potent antioxidant properties (Lee et al., 2009; Almeida Rezende et al., 2016; Hua et al., 2016; Fernandes et al., 2017; Qadir, 2017; Spagnuolo et al., 2018). All of these features of flavonoids make them a promising therapeutic intervention against neurodegenerative diseases. Though several natural products have been shown to have potential epigenetic modulatory properties against cancer and CVDs, very few natural product inhibitors have been shown to modulate the epigenetic pathways in neurological disorders.

Quercetin is a plant flavonoid present in most plants and foods, such as red wine, onions, green tea, apples, berries, Ginkgo biloba, American elder, and others. The molecular mechanisms underlying the neuroprotective actions of quercetin include possible up- and/or down-regulation of cytokines *via* nuclear factor (Nrf2), Paraoxonase-2, c-Jun N-terminal kinase (JNK), Protein kinase C, Mitogen-activated protein kinase (MAPK) signaling cascades, and PI3K/Akt pathways, as demonstrated by *in vivo* and *in vitro* studies (Zaplatic et al., 2019). Cocoa is a rich source of plant flavonoids and shows neuroprotective action against cognitive decline in healthy individuals (Sorond et al., 2008; Lamport et al., 2015). In a clinical trial involving 531 participants aged ≥65 years, chocolate consumption for 48 months was associated with a 41% lower risk of cognitive decline (Moreira et al., 2016). Anthocyanin is a bioactive compound found in the seed coat of the black soybean and is reported to inhibit several diseases. A recent study established that supplementation with anthocyanins mitigates oxidative stress, neurodegeneration, and memory impairment in a mouse model of AD *via* the PI3K/Akt/Nrf2/HO-1 pathways (Ali et al., 2018).

Caffeine reverses cognitive impairment and decreases brain Aβ levels in aged APP mice (Azam et al., 2003; Arendash et al., 2009). This reduction in Aβ plaque might be due to the stimulation of protein kinase A activity, increased phosphor-CREB levels, and reduced phosphor-JNK and phosphor-ERK expression in mouse models of AD and promotes survival cascades in the brain (Zeitlin et al., 2011). Interestingly, higher blood caffeine levels in MCI patients have been linked to a lack of progression to dementia (Cao et al., 2012). A population-based study reported that drinking of 3–5 cups of coffee per day might reduce the incidence of AD and dementia by 65% (Eskelinen et al., 2009). While animal data recommend a protective effect for caffeine on cognition, studies in humans remain inconsistent. A study on 3,494 men showed that coffee and caffeine intake in midlife was not related to cognitive impairment (Gelber et al., 2011). Conflicting results were reported in different populations, wherein a Portuguese study showed an association of caffeine consumption with reduced cognitive decline (Santos et al., 2010), while another study did not show any association in a population in France (Ritchie et al., 2007).

### Resveratrol

Resveratrol, a polyphenol present in grapes and red wine, is receiving increasing attention due to its strong antioxidant and anti-inflammatory actions (Gambini et al., 2015; Sawda et al., 2017). Resveratrol exhibits these properties due to its molecular structure, which endows it with the ability to bind with several biomolecules. Resveratrol is known to activate sirtuin 1 (SIRT1), a class III HDAC (Baur, 2010), and thereby protect cells against the inflammation and oxidative damage induced by ROS (Cantó et al., 2009). Resveratrol activates a transcriptional coactivator, PGC-1α, that promotes energy metabolism by glucose uptake and mitochondrial biogenesis (Lagouge et al., 2006; Kumar and Lombard, 2015; Parihar et al., 2015). Recent studies have demonstrated that maternal resveratrol supplementation and vitamin D combined with resveratrol could prevent cognitive impairment in SAMP8 mice offspring through amyloidogenic pathways, neuroinflammation, tau phosphorylation, epigenetic changes, and cell signaling pathways (Cheng et al., 2017; Izquierdo et al., 2019). Another study indicated the ameliorative action of resveratrol in hippocampal neurodegeneration and memory performance (Gomes et al., 2018). Some clinical trials on supplementation with resveratrol for a longer period reported improved cognitive decline and improved functional connectivity of the hippocampus (Witte et al., 2014). Several clinical trials on resveratrol supplementation and its possible neuroprotective impact on cognitive decline, MCI, and AD are ongoing (Tome-Carneiro et al., 2013). Owing to its strong antioxidant, anti-inflammatory, and neuroprotective properties, supplementation with resveratrol may be a promising therapeutic measure to combat the rising prevalence of cognitive deficit and AD (Cheng et al., 2017).

All of these dietary bioactive compounds, such as curcumin, resveratrol, epigallocatechin-3-gallate, genistein, phenylisothiocyanate, and indole-3-carbinol, have the ability to modulate epigenetic mechanisms including regulation of HDAC and HAT activities and acetylation of histones and non-histone chromatin protein (Vahid et al., 2015).

### Minerals

Deficiency of dietary minerals such as calcium, magnesium, and potassium plays an important role in a wide variety of critical cellular processes associated with cognitive impairment and dementia (Ozawa et al., 2012; Cherbuin et al., 2014). Substantial evidence shows that higher levels of dietary minerals play a protective role against many metabolic diseases including type 2 diabetes, hypertension, stroke, and cognitive decline (Iso et al., 1999; Larsson and Wolk, 2007; Villegas et al., 2009; Barbagallo et al., 2011). Compelling evidence shows that magnesium deficiency may induce oxidative stress in various tissues through a substantial increase in the formation of free radicals by inflammatory cells, which further impairs memory and contributes to AD pathology (Durlach, 1990; Bardgett et al., 2005; Vural et al., 2010; Barbagallo et al., 2011). Previous studies have demonstrated that magnesium supplementation modifies AβPP processing and stimulates the α-secretase cleavage pathway (Yu et al., 2010) and plays a potential protective role in cognitive dysfunction (Cilliler et al., 2007). Further well-designed clinical trial studies are required to ascertain the protective role of magnesium in cognitive decline and AD pathology.

### Vitamin Supplementation

Vitamins perform vital functions in the nervous system and might be useful in maintaining cognitive function and delaying the onset of AD (McCleery et al., 2018). Vitamin supplements are found to be very effective in reducing the burden of chronic diseases, including CVD and cancer. These dietary interventions target various molecular mechanisms in disease pathology, including oxidative stress, mitochondrial dysfunction, inflammatory pathways, and calcium homeostasis, in many diseases. A recent study demonstrated the role of vitamins in aging, MCI, and AD by the modulation of many molecular mechanisms involved in the pathogenesis of the disease (Fenech, 2017). Very few randomized clinical trials have examined the effectiveness of vitamin supplements on the primary prevention of cognitive decline and AD, and contradictory results have been reported from the few clinical studies on dietary interventions in AD. A randomized trial of beta-carotene supplementation and cognitive function in 4,052 men did not show any significant effect on cognitive function (Grodstein et al., 2007). However, mixed results have been reported for vitamin B supplementation in cognitive impairment. A randomized clinical trial of folic acid, vitamin B6, and B12 supplementation by 299 men aged >75 years did not show a significant effect on cognitive function (Ford et al., 2010). Similarly, a meta-analysis of nine RCTs involving 2,835 persons exhibited no significant effect of folic acid with or without other B vitamins on cognitive function (Wald et al., 2010). On the other hand, another study showed that supplementation with folic acid and vitamin B12 together were significantly improving cognitive functions (Walker et al., 2012).

### Mitochondria-Targeted Antioxidants

The excess of ROS produced in cognitive decline and AD is associated with mitochondrial dysfunction, represented by altered biogenesis and dynamics (Calkins et al., 2011). Mitochondria-targeted drugs may be a promising therapeutic strategy in aging and neurodegenerative diseases (Reddy, 2008). In the past decade, many mitochondria-targeted antioxidants have come on the market as supplements for delaying the onset of brain diseases by boosting mitochondrial biogenesis and bioenergetics. These mitochondria-targeted antioxidants are known to improve a variety of pathologic conditions, including heart disease, obesity, diabetes-related complications, and AD by modulating the oxidative stress markers and misfolded proteins (Manczak et al., 2010; Reddy and Reddy, 2011; Bhatti et al., 2017a; Reddy et al., 2017). Some of the mitochondria-targeted antioxidant molecules currently available in the market are MitoQ, MitoVitE, MitoTempo, MitoPBN, and MCAT, which have the potential to limit free radical formation and improve mitochondrial dysfunction in many diseases.

### Mediterranean Diet Pattern

Lifestyle and diet have been identified as major risk factors in a number of diseases. The MD, broadly accepted as a healthy eating model, is characterized by the high consumption of plant-based foods, olive oil as the main source of fat, low-to-moderate consumption of fish, dairy products, and poultry, low consumption of red and processed meat, and low-to-moderate consumption of wine with meals. Earlier studies demonstrated that MD is linked with low morbidity and mortality in several diseases including CVDs, diabetes, obesity, cancer, and neurodegenerative diseases (Roman et al., 2008; Temple et al., 2019; Witlox et al., 2019). These dietary interventions impact several cardiovascular risk determinants, including body weight, blood pressure, and lipid levels (Rees et al., 2019; Temple et al., 2019). Previous studies have shown that higher adherence to the MD may reduce the risk of developing diabetes and CVDs (Esposito et al., 2017). The modulatory action of the MD is mediated through the molecular mechanisms involving inflammation and metabolic abnormalities in AD pathology (Akiyama et al., 2000; Esposito et al., 2004; Scarmeas et al., 2006; Gu et al., 2010). Diet-derived bioactive components modulate DNA methylation by altering histones and chromatin structure (Bassett and Barnett, 2014). Recent studies also suggest that following the Mediterranean dietary pattern may reduce the risk of many types of cancers (Farinetti et al., 2017; Jones et al., 2017; Schwingshackl et al., 2017).

## Lifestyle Modifications

### Physical Activity

A sedentary lifestyle is considered as one of the risk factors for a wide variety of diseases in the 21st century (Blair, 2009). Physical activity is defined as any bodily movement produced by skeletal muscles that result in energy expenditure (Caspersen et al., 1985). Physically active individuals are healthy and free from many diseases (Colberg et al., 2016). Recent studies demonstrated relative reductions of 10% per decade in the prevalence of seven modifiable risk factors per decade might reduce the prevalence of AD in 2050 by 8.3% worldwide (Norton et al., 2014; Luck and Riedel-Heller, 2016). This kind of preventive measure could have a high impact on the burden of lifestyle-related diseases (Ashby-Mitchell et al., 2017). Physical activity is one of the potentially effective training interventions that can limit the prevalence of a wide variety of cardiometabolic and neurodegenerative diseases by reducing mitochondrial dysfunction by activating various transcription factors in bioenergetics processes (Barbieri et al., 2015).

Regular exercise activates various cell signaling pathways and helps improve the mitochondrial health in the skeletal muscles (Russell et al., 2014). It is known to control the blood sugar level and body weight, maintain blood pressure, reduce dyslipidemia, and improve muscles and bone health. Another study demonstrated a reduction in cognitive decline and a decrease in the accumulation of misfolded proteins in the brain of transgenic animals (Pietropaolo et al., 2008). Another study indicated that physical exercise induces neuroplasticity of the brain and improves cognitive functions, as evidenced by animal and human studies (Hötting and Röder, 2013). Physical activity controls the cellular energy homeostasis through PGC-1α and a nicotinamide adenosine dinucleotide (NAD)-dependent deacetylase, SIRT1 (Rodgers et al., 2005). CR or exercise reduces energy and increases the AMP/ATP ratio, which activates 5′-adenosine monophosphate-activated protein kinase (AMPK) in the cells. These events further cause stimulation of a transcription factor, PGC 1, through phosphorylation and then ultimately induce mitochondrial biogenesis (Jäger et al., 2007). With aging, there is a loss of muscle mass and muscle activity. Regular exercise reduces the development of aging-related muscle deterioration and promotes healthy aging (Cartee et al., 2016).

### Calorie Restriction

Calorie restriction (CR) is another potentially promising non-pharmacologic intervention that is effective in brain aging by improving metabolic health (Wahl et al., 2019). CR is effective through neutralizing the harmful effects of ROS and oxidative damage (Barja and Herrero, 2000; Zainal et al., 2000; Barja, 2002; Civitarese et al., 2007). CR has been shown to prevent the development of various diseases through sirtuins as a target. A previous study showed that long-term CR significantly reduces β-amyloid and γ-secretase in female Tg2576 mice (Schafer et al., 2015) and plays a preventive role in AD pathology. Observational trials and RCTs indicate that CR in humans improves multiple metabolic factors that are involved in the pathophysiology of cardiometabolic disorders (Fontana, 2008). CR exerts these modulations by enhancing their properties by inhibiting vital nutrient-sensing and inflammatory pathways (Most et al., 2017). So, as well as physical activity and exercise, CR may also be considered as a promising nutritional intervention for the prevention of many age-related chronic diseases.

## Conclusions and Future Perspectives

The world population is aging at a greater pace, and age-associated diseases are therefore a matter of concern today. The rapid increase in the incidence and prevalence of dementia globally is a major problem and is linked with social and economic burdens. Unfortunately, there is no permanent cure available to date. Several pharmacological therapeutics targeting the major molecular mechanisms, including Aβ and tau proteins, have failed to achieve satisfactory results in human clinical trials. There is thus an urgent need to slow down cognitive decline and halt the progression to AD. Non-pharmacologic interventions such as lifestyle and nutritional therapies, which have been proved to be beneficial in the many aging-related metabolic diseases including diabetes, obesity, CVD, and cancer, may help in the prevention of dementia. The unsatisfactory results of some of the clinical trials may be due to methodological heterogeneity among different studies. These non-pharmacologic interventions, if implemented carefully, may be very effective in reducing the exponential rise in the incidence of AD and curtailing the economic burden on the individuals affected and society. The area that needs great attention is the molecular mechanisms and effective therapeutic targets for AD. Also, large randomized clinical trials should be carried out to ascertain the effectiveness of new drugs or non-pharmacologic interventions for cognitive deficit and AD in the human population. The next decade will be a critical period for the scientific community to discover an effective treatment for AD.

## Author Contributions

GB, JB, AR, and PR contributed for planning, execution, writing and final drafting of the manuscript.

## Conflict of Interest

The authors declare that the research was conducted in the absence of any commercial or financial relationships that could be construed as a potential conflict of interest.
